# Genome-wide Identification and analysis of the stress-resistance function of the TPS (Trehalose-6-Phosphate Synthase) gene family in cotton

**DOI:** 10.1186/s12863-016-0360-y

**Published:** 2016-03-18

**Authors:** Min Mu, Xu-Ke Lu, Jun-Juan Wang, De-Long Wang, Zu-Jun Yin, Shuai Wang, Wei-Li Fan, Wu-Wei Ye

**Affiliations:** State Key Laboratory of Cotton Biology / Institute of Cotton Research of Chinese Academy of Agricultural Sciences, Anyang, Henan 455000 China

**Keywords:** Rehalose-6-phosphase synthase (TPS), *Gossypium raimondii*, *Gossypium arboretum*, *Gossypium hirsutum* L, Gene family

## Abstract

**Background:**

Trehalose (a-D-glucopyranosyl a-D-glucopyranoside) is a nonreducing disaccharide and is widely distributed in bacteria, fungi, algae, plants and invertebrates. In the study, the identification of trehalose-6-phosphate synthase (*TPS*) genes stress-related in cotton, and the genetic structure analysis and molecular evolution analysis of *TPSs* were conducted with bioinformatics methods, which could lay a foundation for further research of *TPS* functions in cotton.

**Results:**

The genome information of *Gossypium raimondii* (group D), *G. arboreum* L. (group A), and *G. hirsutum* L. (group AD) was used in the study. Fifty-three *TPSs* were identified comprising 15 genes in group D, 14 in group A, and 24 in group AD. Bioinformatics methods were used to analyze the genetic structure and molecular evolution of *TPSs*. Real-time PCR analysis was performed to investigate the expression patterns of gene family members. All *TPS* family members in cotton can be divided into two subfamilies: Class I and Class II. The similarity of the *TPS* sequence is high within the same species and close within their family relatives. The genetic structures of two *TPS* subfamily members are different, with more introns and a more complicated gene structure in Class I. There is a TPS domain(Glyco transf_20) at the N-terminal in all *TPS* family members and a TPP domain(Trehalose_PPase) at the C-terminal in all except *GrTPS6*, *GhTPS4*, and *GhTPS9*. All Class II members contain a UDP-forming domain. The responses to environmental stresses showed that stresses could induce the expression of *TPSs* but the expression patterns vary with different stresses.

**Conclusions:**

The distribution of *TPS*s varies with different species but is relatively uniform on chromosomes. Genetic structure varies with different gene members, and expression levels vary with different stresses and exhibit tissue specificity. The upregulated genes in upland cotton TM-1 is significantly more than that in *G. raimondii* and *G. arboreum* L. *Shixiya* 1.

**Electronic supplementary material:**

The online version of this article (doi:10.1186/s12863-016-0360-y) contains supplementary material, which is available to authorized users.

## Background

**Fig. 1:**
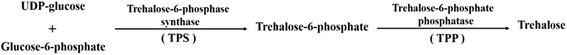
Trehalose biosynthesis pathway in plant

Trehalose, a non-reducing disaccharide, is composed of two glucose molecules that are connected by α, α-1, 1-glycosidic linkage and exist in bacteria, fungi, algae, invertebrates, and plants [1]. Trehalose protects bioactive substances and cell structures, such as proteins, nucleic acids, and biological membranes, under adverse environmental stresses, such as drought, freezing, oxidation, high salt, high temperature and low temperature [1–4]. Trehalose synthesis in plants is a two-step process: first, trehalose-6-phosphase synthase (TPS) catalyzes UDP-glucose and glucose-6-phosphate to generate trehalose-6-phosphate (T6P); second, trehalose-6-phosphate phosphatase (TPP) catalyzes the dephosphorylation of trehalose-6-phosphate to trehalose (Fig. 1). The structure of *TPS* proteins in plants contains two domains: TPS and TPP; however, many studies have shown that the TPP domain in TPS proteins appears to have lost enzymatic activity during evolution [5, 6].

Blaquez et al. [7] screened the *Arabidopsis* cDNA library and, for the first time, a *TPS* gene was cloned named as *AtTPS1* which had the trehalose-6-phosphase synthetase function from higher plants [7]. The *AtTPS1* mutant *TPS1* was a recessive embryonic lethal gene [8]. Even so, *AtTPS1* played an important role in the process of vegetative growth and transition to flowering [9, 10]. Zentella et al. [11] cloned *TPS* from *Selaginella,* named *SlTPS1*, and the study found that *SlTPS1* maintained the biosynthesis of trehalose and played an important role in responding to heat and salt stress [11]. Studies showed that *TPS* expression levels in cotton increased under drought stress [12] and *TPS* genes in maize were also found upregulated in response to both salt and temperature stress [13]. *OsTPS1* might enhance the abiotic stress tolerance of rice by increasing the trehalose and proline content [14]. Many studies have suggested that *TPSs* play a vital role in plants adjusting to environmental stresses.

Higher plants comprise a series of *TPS* gene families [15]. The *Arabidopsis TPS* gene family contains 11 members(*AtTPS1-11*) [5], while rice contains 11 members (*OsTPS1-11*) [16], poplar contains 12 members (*PtTPS1-12*) [6], and 28 genes for *TPS*s are found from Pigeon pea [17]. However, cotton *TPS* has not yet been systematically researched.

Cotton is an important economic and oil crop, a model plant for the study of plant polyploidy, cell wall biosynthesis, and cell elongation [18]. Cultivated cottons include both diploid and tetraploid species. The diploid cotton *Gossypium raimondii* D5 (group D) and the *G. arboreum* L. A2-8 (group A) and the tetraploid cotton *G. hirsutum* L. TM-1 were sequenced over the years [19–21]. This study investigated the distribution of *TPSs* from whole genome-wide and genetic structure of *TPS* genes in three cotton genomes, and examined the expression patterns of the gene family members in different tissues under different stresses. And the results were important for the study of stress-resistance mechanism and the improvement of adversity-resistance in cotton.

## Results

### Genome-wide identification of cotton *TPS* family members

With the *GaTPS1* sequence (Accession No.: EU750912.1) and *AtTPS1* sequence (Accession No.: XM_002889154.1) as references, local BLAST analysis was performed based on genomes data of *G. raimondii*, *G. arboreum* L., and upland cotton TM-1. Fourteen *TPS*s were found in group A, 13 genes of which were named *GaTPS*2–*GaTPS*14 according to their sequence in the chromosome; *GaTPS*1 was not included. Fifteen *TPS*s were found in group D and named *GrTPS*1–*GrTPS*15 according to their sequence in the chromosome. 24 *TPS*s in group AD were named *GhTPS*1–*GhTPS*24, respectively (Table 1). *TPS* contained from 98 to 1109 amino acid residue numbers (AA), but most contained between 800 and 1000. *GhTPS*4 contained 544 amino acids; *GhTPS*9 contained only 98. This might be associated with gene domain differences. The isoelectric point (PI) ranged from 4.59 to 8.04. The protein molecular weight ranged from 10.76 to 130.28 kDa. The subcellular localization prediction showed that most *TPS*s were located in cytoplasm but some of which were located on the cytoplasmic membrane and in the nucleus.

**Table 1:** Basic characteristic of *TPS* genes in cotton genome

Gene	Accession number	CDS	AA	pI	Mw	Predicted subcellular localization
name		(bp)			(kDa)	
GaTPS1	EU750912.1	2586	861	6.27	96.96	Cytoplasmic
GaTPS2	Cotton_A_09724	2589	862	5.74	97.6	Cytoplasmic
GaTPS3	Cotton_A_38312	2517	838	5.74	95.55	Cytoplasmic
GaTPS4	Cotton_A_22808	2610	869	5.75	98.49	Nuclear
GaTPS5	Cotton_A_05024	2574	857	5.94	97.22	Cytoplasmic
GaTPS6	Cotton_A_31871	2829	942	6.91	106.42	Cytoplasmic
GaTPS7	Cotton_A_22769	2829	942	6.43	106.17	Cytoplasmic
GaTPS8	Cotton_A_02720	2562	853	5.63	96.39	Plasma Membrane
GaTPS9	Cotton_A_17512	2574	857	5.78	96.64	Cytoplasmic
GaTPS10	Cotton_A_12395	2574	857	5.70	96.88	Nuclear
GaTPS11	Cotton_A_12382	2952	983	6.33	111.4	Cytoplasmic
GaTPS12	Cotton_A_23712	2754	917	5.66	104.05	Cytoplasmic
GaTPS13	Cotton_A_23709	2652	883	6.58	99.37	Cytoplasmic
GaTPS14	Cotton_A_09181	2595	864	5.75	97.4	Cytoplasmic
GrTPS1	Cotton_D_gene_10022693	2562	853	5.84	96.33	Plasma Membrane
GrTPS2	Cotton_D_gene_10020837	2808	935	6.70	105.47	Cytoplasmic
GrTPS3	Cotton_D_gene_10038561	2517	848	5.74	95.54	Cytoplasmic
GrTPS4	Cotton_D_gene_10020702	2754	917	5.67	104.01	Cytoplasmic
GrTPS5	Cotton_D_gene_10020699	3507	1168	6.39	130.28	Plasma Membrane
GrTPS6	Cotton_D_gene_10020701	2517	838	8.04	93.69	Plasma Membrane
GrTPS7	Cotton_D_gene_10033185	2595	864	5.77	97.6	Cytoplasmic
GrTPS8	Cotton_D_gene_10037806	2589	862	5.8	97.67	Cytoplasmic
GrTPS9	Cotton_D_gene_10033657	2574	857	5.66	96.84	Cytoplasmic
GrTPS10	Cotton_D_gene_10033644	2886	961	6.27	108.94	Cytoplasmic
GrTPS11	Cotton_D_gene_10000478	2517	838	5.89	94.92	Cytoplasmic
GrTPS12	Cotton_D_gene_10031457	2574	857	5.7	96.53	Cytoplasmic
GrTPS13	Cotton_D_gene_10023754	2610	869	5.7	98.48	Nuclear
GrTPS14	Cotton_D_gene_10006852	2829	942	6.21	105.94	Cytoplasmic
GrTPS15	Cotton_D_gene_10009065	2586	861	6.24	96.88	Cytoplasmic
GhTPS1	CotAD_09030	2673	890	5.9	100.86	Plasma Membrane
GhTPS2	CotAD_24641	2610	869	5.7	98.489	Nuclear
GhTPS3	CotAD_51660	2574	857	5.85	96.64	Cytoplasmic
GhTPS4	CotAD_66147	1635	544	6.39	61.95	Cytoplasmic
GhTPS5	CotAD_22604	2826	941	5.71	106.63	Cytoplasmic
GhTPS6	CotAD_22606	2652	883	6.41	99.349	Cytoplasmic
GhTPS7	CotAD_03946	2595	864	5.82	97.57	Cytoplasmic
GhTPS8	CotAD_16380	2634	877	6.42	98.72	Cytoplasmic
GhTPS9	CotAD_16379	297	98	4.59	10.76	Extracellular
GhTPS10	CotAD_25711	2574	857	5.61	96.84	Nuclear
GhTPS11	CotAD_21592	2829	942	6.3	106	Cytoplasmic
GhTPS12	CotAD_25696	2808	935	6.83	106.28	Nuclear
GhTPS13	CotAD_66378	2517	838	5.7	95.58	Cytoplasmic
GhTPS14	CotAD_16567	2589	862	5.7	97.66	Cytoplasmic
GhTPS15	CotAD_52287	2952	983	6.27	111.48	Cytoplasmic
GhTPS16	CotAD_53302	2286	761	5.75	86.67	Cytoplasmic
GhTPS17	CotAD_74149	2559	755	6.27	85.75	Cytoplasmic
GhTPS18	CotAD_51342	2574	857	5.7	96.91	Nuclear
GhTPS19	CotAD_05656	2574	857	5.86	96.97	Cytoplasmic
GhTPS20	CotAD_00585	2574	857	5.78	96.57	Cytoplasmic
GhTPS21	CotAD_17819	2562	853	5.67	96.34	Plasma Membrane
GhTPS22	CotAD_24751	2808	935	6.7	105.44	Cytoplasmic
GhTPS23	CotAD_36474	2589	862	5.85	97.73	Cytoplasmic
GhTPS24	CotAD_40190	2808	935	6.8	105.57	Cytoplasmic

### Multiple sequence alignment and phylogenetic analysis of *TPS* in cotton

**Fig. 2:**
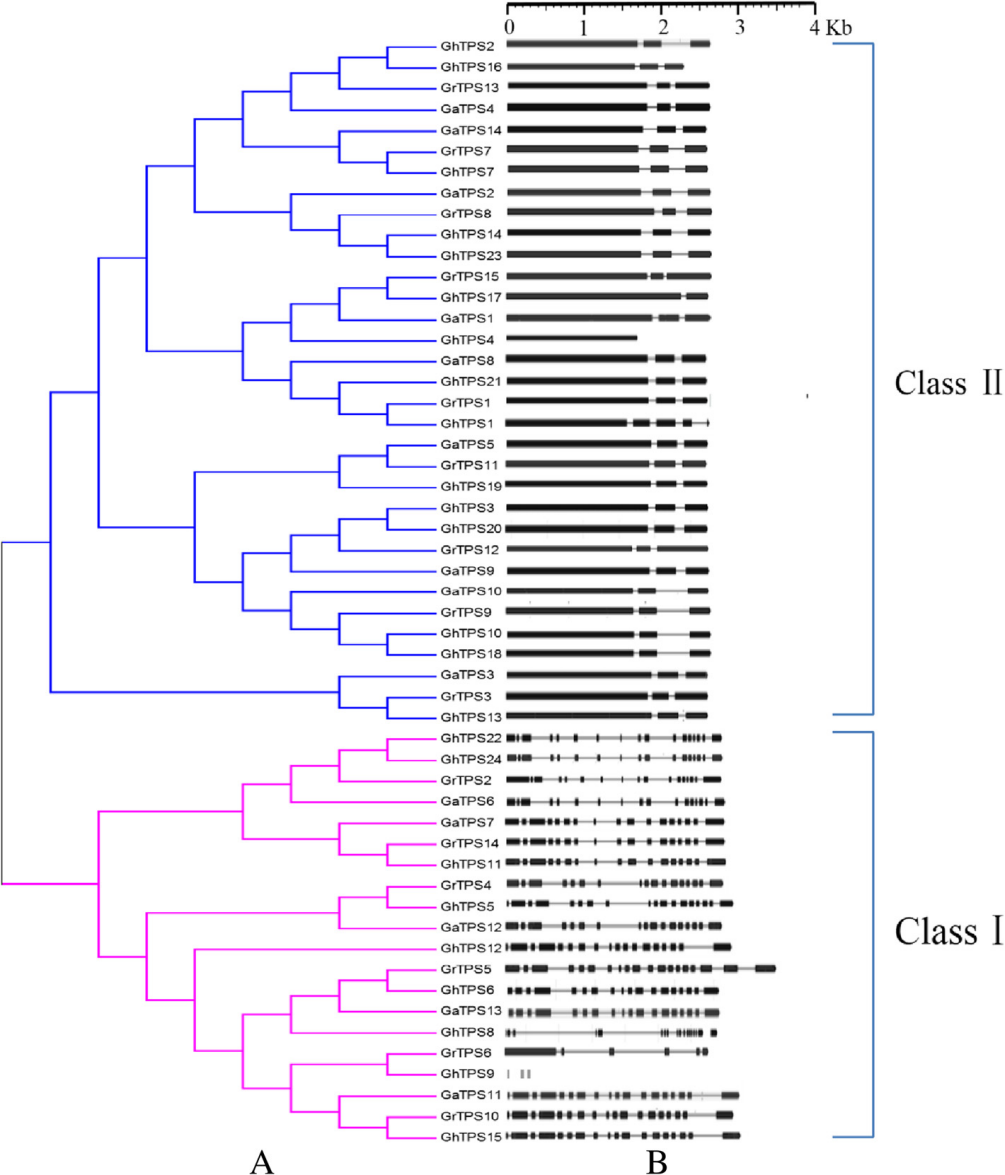
Phylogenetic analysis (**a**) and gene structure (**b**) of the *TPS* gene family in cotton. The cotton *TPS* family members were divided into two subfamilies, Class I and Class II. Exons Introns

To assess the *TPS* evolutionary relationship of *G. raimondii*, *G. arboreum* L., and *G. hirsutum* L., multiple sequence alignment of 53 *TPS* family members was conducted (Additional file 1) and the evolutionary tree was constructed (Fig. 2a). According to the evolutionary tree, the cotton *TPS* family members were divided into two subfamilies, Class I and Class II. As shown in Fig. 2a, Class I contains 20 members, comprising 6 in *G. raimondii* group D, 5 in *G. arboreum* L. group A, and 9 in upland cotton group AD. Class II contains 33 members, comprising 9, 9, and 15 members in groups D, A, and AD, respectively.

### Gene structures and protein domains of cotton *TPSs*

Gene structure analysis is an important method by which to study genetic evolution. The numbers of introns and exons in *TPS* family members were calculated in *G. raimondii*, *G. arboreum* L., and *G. hirsutum* L. and the *TPS* structure in cotton was created (Fig. 2b). The result showed that, except for *GrTPS5*, *GhTPS4*, and *GhTPS9,* the code length of the remaining 50 family members ranged from 2500 to 3000 bp. Not any big difference occurred in gene length; however, the difference in the genetic structure of *TPSs* was significant between the two subfamily members, where the number of gene introns was larger and the genetic structures were more complicated in Class I than in Class II.

The domain analysis showed that, with the exception of *GrTPS6*, *GhTPS4*, and *GhTPS9*, a TPS structure domain (Glyco_transf_20) in 53 cotton *TPS* family members is located at the N-terminal and a TPP domain (Trehalose_PPase) at the C-terminal. *GrTPS6*, *GhTPS4*, and *GhTPS9* contain only the TPS domain.

**Fig. 3:**
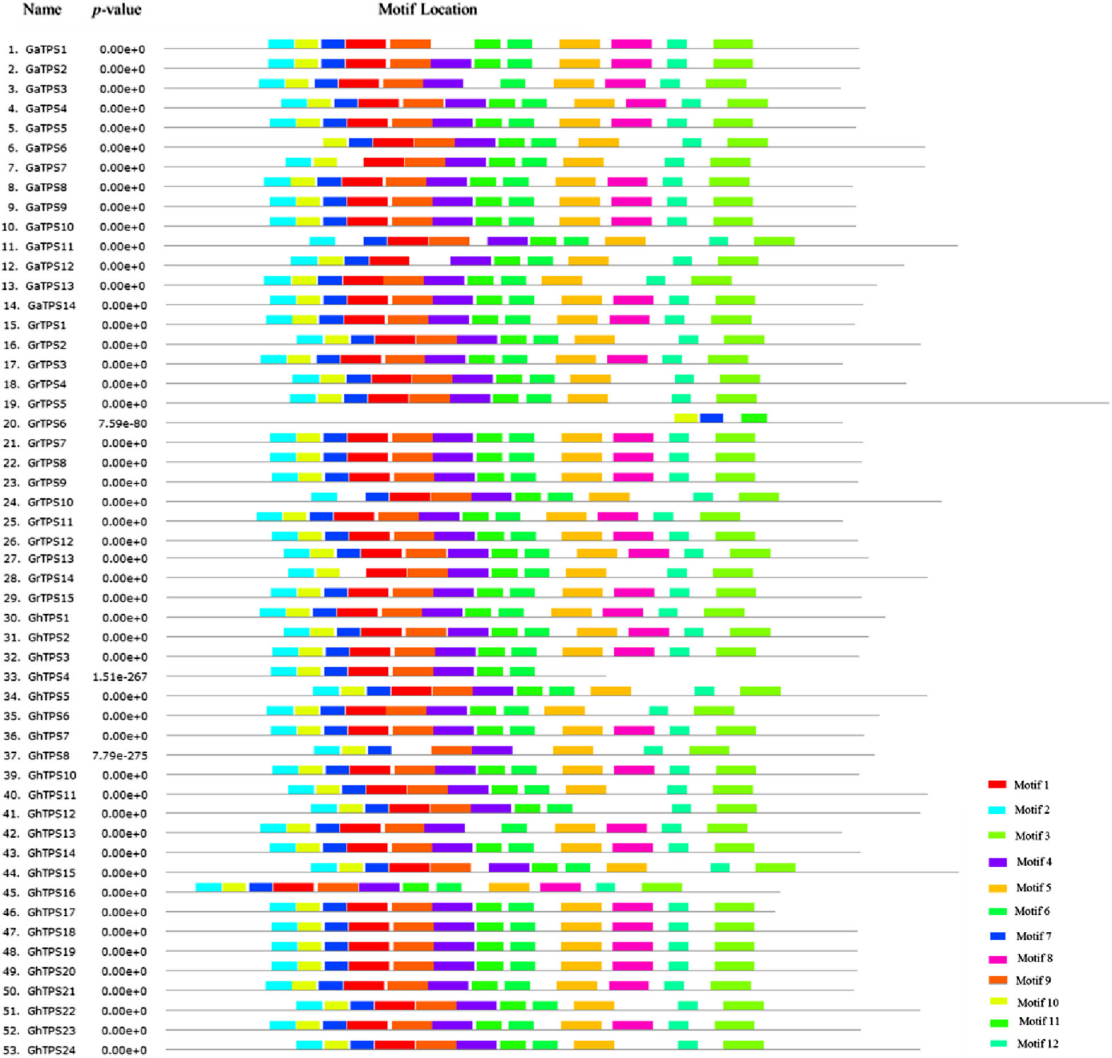
Motif analyse of *TPS* gene family in cotton. A total of 12 putative conserved motifs of cotton TPS proteins is identified using the MEME online program. Motifs 1, 2, 4, 5, 6, 7, 9, 10, and 11 together compose the *TPS* domain (Glyco_transf_20). Motifs 3 and 12 compose the HAD-like domain (TPP domain). Motif 8 has a UDP-forming domain function

Motif analysis of 53 family members is shown in Fig. 3. Cotton *TPSs* totally contain 12 motifs (Additional file 2: Figure S1). Among them, motifs 1, 2, 4, 5, 6, 7, 9, 10, and 11 together compose the TPS domain (Glyco_transf_20). Motifs 3 and 12 compose the HAD-like domain (TPP domain). But motif 8 has a UDP-forming domain that functions separately. It was found that motifs 1, 3, 5, 6, and 12 in group D are conservative, while only motif 11 is conservative in group A. Motif 2 was observed in all group AD members. *GrTPS6* contains an incomplete TPS domain: only motifs 7, 10, and 11. *GhTPS4* contains all TPS domains except for motif 5. *GhTPS9* contains only incomplete motif 2, which is not listed in the Fig. 3. It was speculated that this might be caused in the long evolutionary process. In addition, 20 among the 53 genes without motif 8 are in perfect accord with Class I members in the evolutionary tree (Fig. 2a).

### Distribution and duplication analysis of *TPS* family members

**Fig. 4:**
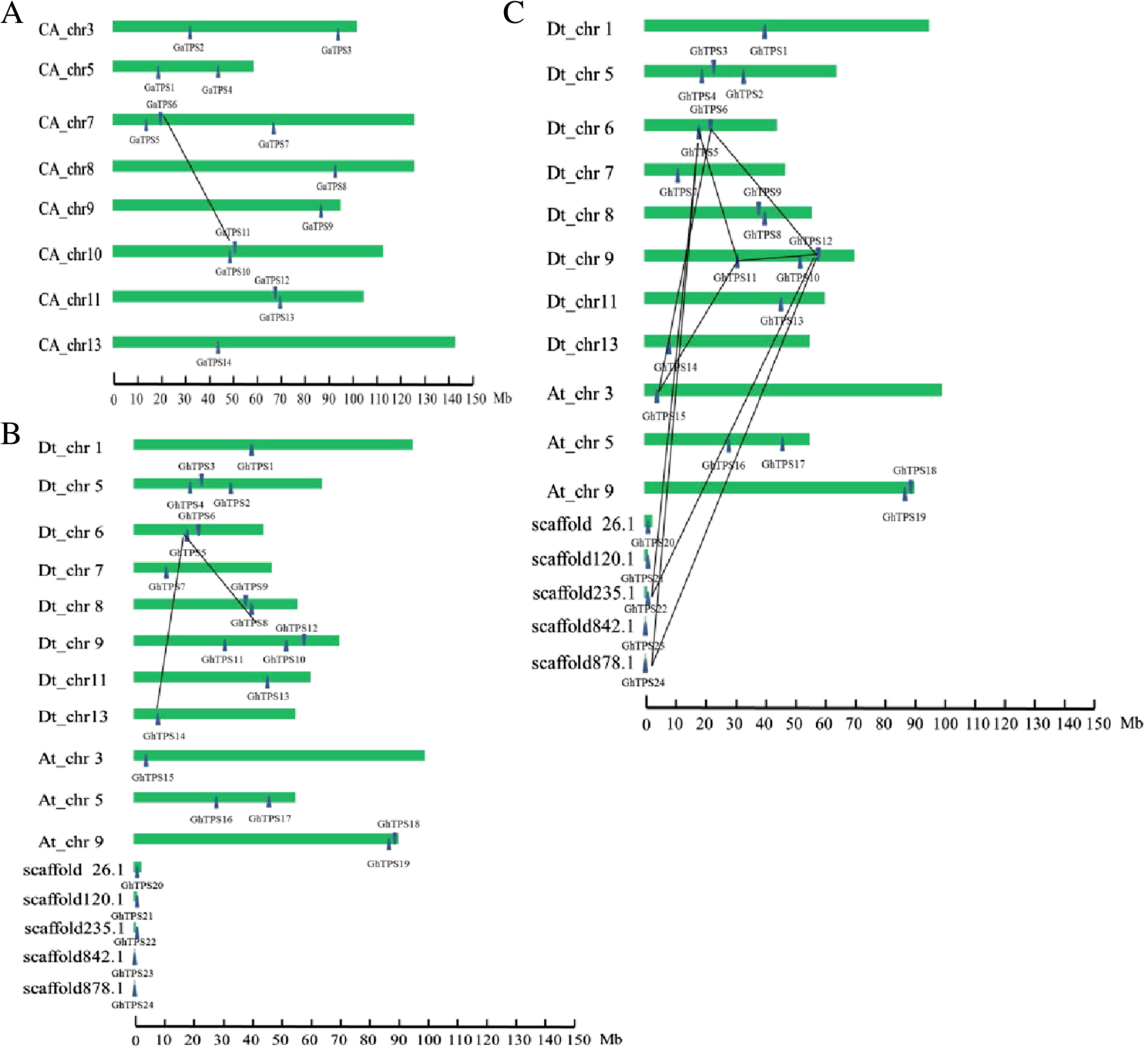
Localization of *TPS*s in the cotton genome. Fifty-three *TPS*s were mapped on different chromosomes in the cotton A-genome (**a**), D-genome (**b**) and AD-genome (**c**). The chromosome numbers are indicated above and some *TPS*s are not located on the corresponding chromosome but on scaffold. Black lines represent the segmental duplications between genes

Gene distribution on the chromosome could provide an important basis for the study of the genes evolution and functions. Combined with the chromosome information on three cotton reference genomes and the *TPS* locations, the cotton *TPSs* distribution map on chromosomes can be drawn (Fig. 4). *G. arboreum* L. has 14 *GaTPS*s, which are located on total 8 chromosomes. There are two genes on chromosomes 3, 5, 10, and 11, separately and only one gene is located on chromosome 8, 9, and 13, separately. However, the rest of three genes are located on chromosome 7 (Fig. 4a). Among the 15 *GrTPS*s from *G. raimondii*, three are located on chromosome 6, two on chromosome 9, and one each on chromosomes 1, 2, 4, 7, 8, 10, 11, 13. The remaining two genes are not located on a chromosome—*GrTPS14* is located on scaffold 254 and *GrTPS15* on scaffold 321 (Fig. 4b). Five *TPS*s in upland cotton are not located on the corresponding chromosome but on scaffold 26.1, 120.1, 235.1, 842.1, and scaffold 878.1, respectively. The remaining 19 genes from subgroups A and D are unevenly distributed. In subgroup A, two genes are located on chromosome 5, two on chromosome 9, and 1 on chromosome 3. In subgroup D, three genes are located on chromosomes 5 and 9 separately, two on chromosomes 6 and 8, and each of the remaining genes on chromosomes 1, 7, 11, and 13, respectively (Fig. 4c).

Gene duplications in genomes could provide important information for gene evolution analysis. In the research, we performed gene duplication analysis in genome A, genome D and genome AD, respectively (Fig. 4). Usually, the criteria for inferring a gene duplication event are (1) the length of the alignment sequence covers ≥75 % of the longest gene, and (2) the similarity of the aligned regions is ≥70 % [22, 23]. We totally found 12 gene pairs may be associated with gene duplications. One is in genome A (*GaTPS6/11*), and one is in genome D (*GrTPS5/14*) and others are in genome AD. This characteristic in the same genome is important for gene divergence. And the similar gene structure and gene functions in each gene family may be the result of gene expansion from ancient paralogs or multiple origins of gene ancestry [24]. It was reported that partial fragment replication of the chromosome regions might lead to scattered distribution of gene family members on several chromosomes [25]. Compared with other eukaryotes, plants have a higher rate of gene replication [26] and this might cause an uneven distribution of *TPS* family members. It was reported that whole genome replication occurs in *G. raimondii* at least twice*.* Currently, 2355 linear modules and 39 triple replication regions have been identified [19] and gene duplication and post-separation phenomenon are the two main driving forces of evolution [27, 28].

### Cotton *TPS* family relationships with other plant *TPS*s

**Fig. 5:**
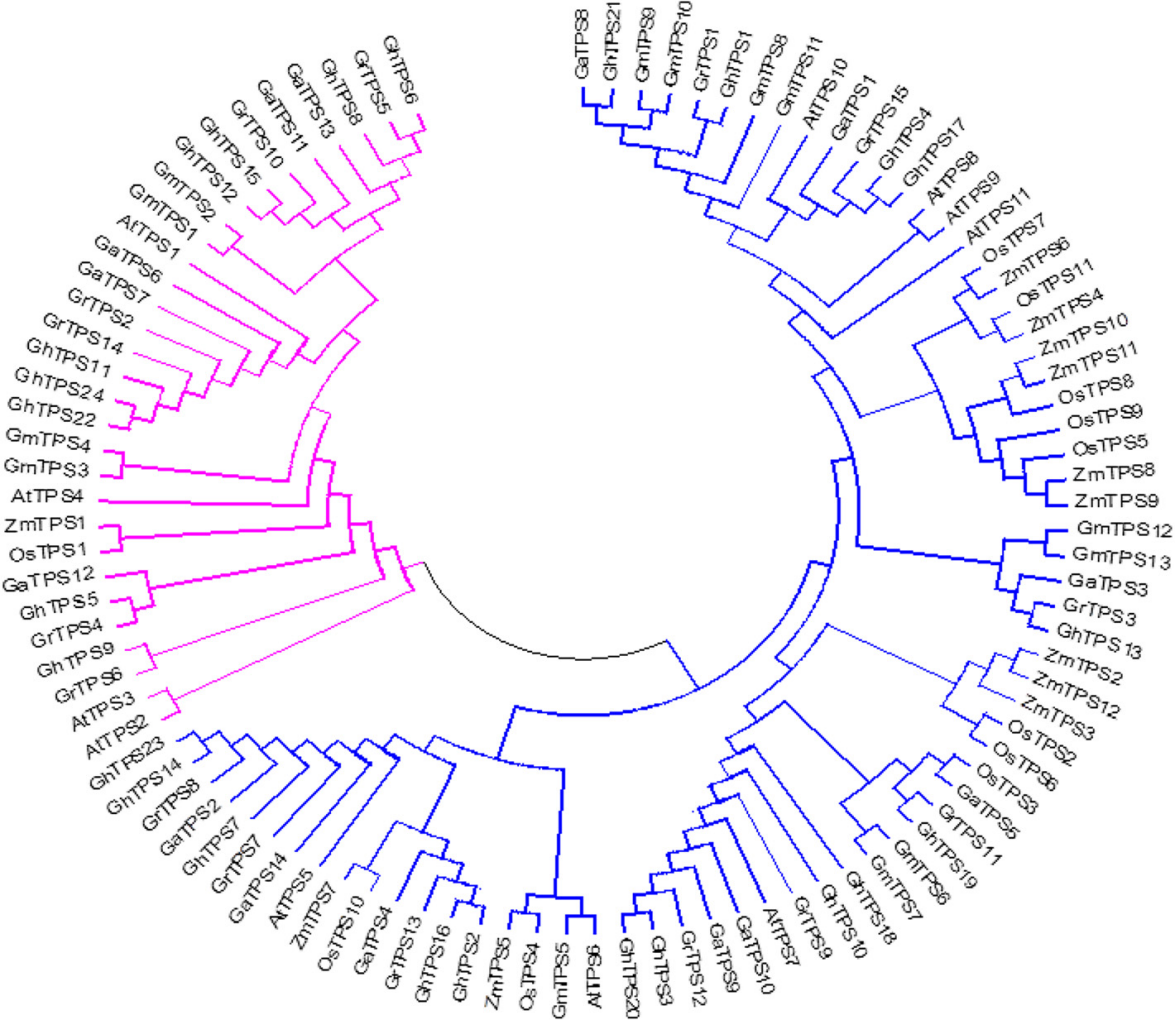
Phylogenetic analysis of the *TPS* gene family in cotton and other plants. The joint unrooted phylogenetic tree containing 14 *G. raimondii* (*GrTPS*), 13 *G. arboreum* L. (*GaTPS*), 24 *G. hirsutum* L. (*GhTPS*), 11 *Arabidopsis* (*AtTPS*),11 rice (*OsTPS*), 13 soybeans (*GmTPS*) and 12 *Zea mays*(*ZmTPS*) *TPS* genes was constructed using neighbor-joining methods

A phylogenetic tree was used to reveal homologous relationships and evolutionary roots of *TPS* from different species. To reveal the evolutionary relationship between the cotton *TPS* family members and those from *Arabidopsis*, rice, *Zea mays*, and soybeans, the amino acid sequence alignment of all members from those species was conducted. MEGA 5.1 was used to construct the phylogenetic tree (Fig. 5). The results showed that compared with the other four species, the relative coefficient of *TPS* from three cotton genomes is higher, indicating a closer relationship. *GrTPS2* and *GrTPS14* of *G. raimondii*, *GaTPS6* and *GaTPS7* of *G. arboreum* L., and *GhTPS11*, *GhTPS22*, and *GhTPS24* of upland cotton have a closer relationship with *TPS1* of Arabidopsis, which suggests that they have similar functions. In addition, parts of the cotton and soybean *TPS* family members are grouped together and show relatively close evolutionary relationships.

### Cotton *TPS* expression pattern analysis under different stresses

**Fig. 6:**
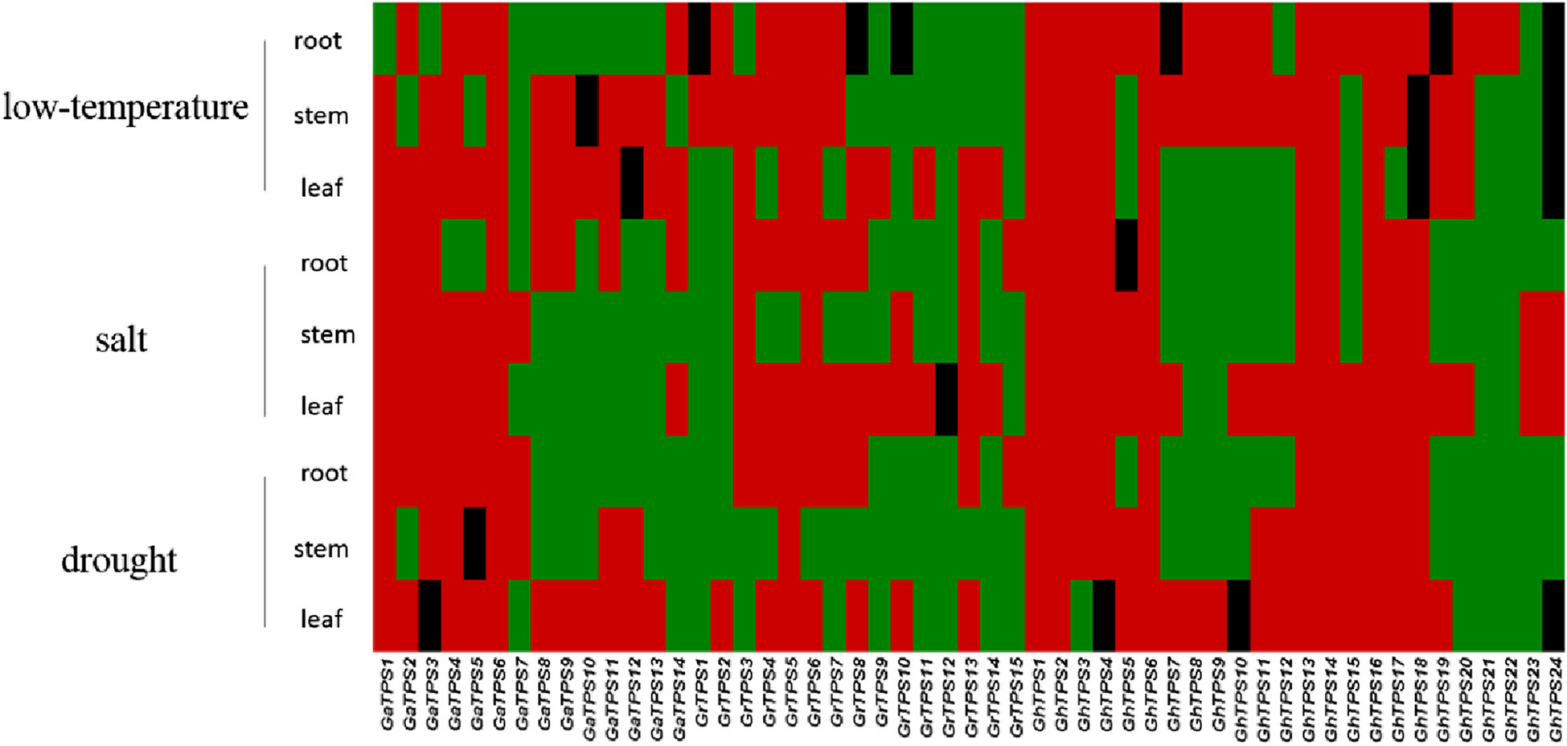
Expression patterns of *TPS* gene family under conditions of cold, salt and drought stress. Quantitative RT-PCR analysis of the expression level of *TPS*s in *G. raimondii*, *G. arboreum* L. *Shixiya* 1, and *G. hirsutum* L. TM-1 including roots, stems and leaves. Results were normalized using *Histone-3* (Accession No. AF02471) as the internal control. Red represents genes upregulated; black represents genes which had no significant change in expression; green represents genes downregulated

To study the expression patterns of *TPS* family members in different tissues under low temperature, drought, and salt stress, *G. raimondii*, *G. arboreum* L. *Shixiya* 1, and *G. hirsutum* L. TM-1 were cultivated at trefoil stage. Real-time quantification PCR after different stresses was conducted. The results showed that most of *TPS* family members expressed in three tissues, including roots, stems, and leaves, and the gene expression levels changed after treatments (Fig. 6). After being exposed to 4.0 °C for 24 h, 6 genes in the roots were upregulated and 8 genes were downregulated in *G. arboreum* L., 5 genes upregulated and 7 genes downregulated in *G. raimondii*, and 19 genes upregulated in upland cotton. 9, 7, and 17 genes in stem were upregulated in groups A, D, and AD, respectively. 12 genes in leaves upregulated in group A, 8 upregulated in group D, and 10 upregulated while 12 downregulated in group AD.

After exposure to 150 mM NaCl, 8, 8, and10 genes in roots upregulated in the three genome groups, respectively. In stems, 7 genes upregulated and 7 downregulated in group A, 4 upregulated and 11 downregulated in group D, and 13 upregulated and 11 downregulated in group AD. In leaves, three and four genes downregulated in group D and group AD, respectively, and seven downregulated in group A.

After being exposed to drought stress, 7 genes in roots upregulated and 7 downregulated in group A, 8 upregulated and 7 downregulated in group D, and 11 upregulated and 13 genes downregulated in group AD. In stems, 7 genes upregulated significantly in group A, only the *GrTPS5 ex*pression level increased in group D, and the expression level of 10 genes increased in group AD. In leaves, 11, 7, and 16 genes upregulated expressed in the 3 groups, respectively.

After exposure to low temperature, salt, or drought, the expression level of *GrTPS12* in group D remained constant in the roots, stems, and leaves. The remaining genes were downregulated (Fig. 6). The expression level of *GaTPS6* in group A and *GhTPS1*, *GhTPS2, GhTPS6*, *GhTPS13*, *GhTPS14*, and *GhTPS16* in group AD significantly increased. It was speculated that these genes in *G. arboreum* L., Shixiya 1 and upland cotton TM-1 played important roles in stress tolerance.

## Discussion

With the increasing research in genomes, comparative genomics methods are used to study gene families, which is one of the hot research topics for several species. Many gene families in different species were identified, such as soybean *LEA* [29], tomato *LBD* [30], *MAPK* [31], and cotton *MAPKKK* [32]. Cotton was one of the pioneer plants in the saline-alkali. *TPS* is closely related to stress resistance and the study of *TPS* from the whole genome would be very important for cotton breeding and the research of stress-resistance mechanism in cotton.

This research investigated *G. raimondii*, *G. arboreum* L., and the upland cotton with the *GaTPS1* sequence (Gene Bank No.: EU750912.1) and *AtTPS1* sequence (Accession No.: XM_002889154.1) as the reference sequences. And totally 53 family members were identified including 15 in group D, 14 in group A, and 24 in group AD, which indicated TPS genes in AD genome were more than that in both A genome and D genome, but not the sum of two genomes. This may be associated with the gene duplications in the evolution of AD genome from their Diploid ancestors. Gene duplication analysis showed 12 gene pairs probably were linked with gene duplication, which was important for their functions research.

Cotton *TPS*s can be divided into two families-20 genes in Class I and 33 genes in Class II, which was consistent with previous studies in *Arabidopsis*, rice, and *Populus* [16]. Domains analysis showed that there is a TPS domain (Glycotransf_20) in all cotton *TPS* members at the N-terminal, while a TPP domain (Trehalose_PPase) at the C- terminal in most *TPS* genes. Functional domains analysis suggested that these domains might be important for *TPS* functions. The process and specific function of each domain involved in regulating function and metabolic pathways remains to be determined with additional studies.

Trehalose could protect bioactive substances and cell structures, such as proteins, nucleic acids, and biological membranes, under adverse environmental stresses, such as high salt, drought, high temperature, freezing, and oxidation [1–4]. In this study, after exposing *G. raimondii*, *G. arboreum* L., *Shixiya 1*, and upland cotton TM-1 to low temperature (4.0 °C), salt (150 mM NaCl), and drought stress (sand moisture content 5.0 %), it was found that, except for *GaTPS6* in group A and *GhTPS1*, *GhTPS2*, *GhTPS6*, *GhTPS13, GhTPS14*, and *GhTPS16* in group AD, the expression levels of the remaining 46 family members varied greatly in different tissues. In group A, *GaTPS3* expression levels in stems and leaves increased after exposure to low temperature and salt, but decreased in roots after exposure to low temperature and remained constant in leaves after exposure to drought. In group D, the expression level of *GaTPS14* in leaves increased after exposure to low temperature and salt, while that of other genes decreased. In group AD, the expression level of *GaTPS18* didn’t change obviously in stems and roots after exposure to low temperatures but increased in each tissue after other stress treatments. The expression level of *GaTPS22* decreased in different tissues after stress treatments, with the exception of increased in roots after exposure to low temperatures. It was found that the rates of upregulated genes in different tissues in upland cotton TM-1 were mostly more than that in *G. raimondii* and *G. arboreum* L. *Shixiya 1*, which also may be associated the stronger tolerance of upland cotton TM-1 to various stresses compared with two diploid cottons.

*TPS* has been found in many plants [5, 6, 12, 13, 33]. The drought tolerance of *Arabidopsis* with overexpressed *AtTPS1* has significantly improved [34]. *OsTPS1* could improve rice-seed tolerance to low temperature, salt, and drought [14]. In this study, the expression levels of *GrTPS5* and *GhTPS15* in different tissues increased under drought stress, which agrees with the results of studies on *Arabidopsis. GrTPS5* expression levels increased under low temperature stress. *GhTPS15* expression levels also increased in roots under low temperature stress and in leaves under salt stress. *GaTPS6* and *GhTPS6* expression levels increased under low temperature, salt, and drought stress, which was in accord with that of rice *OsTPS*1. Previous studies showed that overexpression of *AtTPS1* and *OsTPS*1 in *Arabidopsis* and rice caused phenotypic changes, plant height reduction, and late blooming [9, 10]. It was speculated that overexpression of *GrTPS*5, *GaTPS*6, *GhTPS*6, and *GhTPS*15 in cotton also caused the same phenotypic changes. In addition, it was speculated that the four genes played important roles in cotton against stresses according to their expression levels under stress conditions. Previous reports have shown that *GaTPS*1 is closely related to drought resistance of *G. arboreum* L. [12]. In this study, the expression level of *GaTPS*1 increased significantly in roots, stems, and leaves under drought stress. And also increased in stems and leaves under low temperature stress.

## Conclusions

In this study, totally 53 *TPS* genes in total were identified, including 15, 14 and 24 in group D, group A and group AD, respectively. All *TPS* gene members except *GrTPS6*, *GhTPS4*, and *GhTPS9*, contain a TPS domain(Glyco transf_20) at the N-terminal in *TPS* family members and a TPP domain(Trehalose_PPase) at the C-terminal. Most *TPS* genes could be induced by different stresses, including drought, salt and low temperature, revealing that *TPS* genes may play a vital role in response to stresses. The study lays a foundation for the study of *TPS* functions and the research of cotton growth and development.

## Methods

### Identification of cotton *TPS* family members

The local BLAST was conducted with the *GaTPS1* (Gene Bank No. : EU750912.1) sequence and *AtTPS1* gene sequence (Gene Bank No. : XM_002889154.1) as reference genes in diploid cotton *G. raimondii* (DD) and *G. arboreum* L.(AA) and a tetraploid cotton *G. hirsutum* L. (AADD) genomes. All genomes data of cotton were derived from the Institute of Cotton Research, Chinese Academy of Agricultural Sciences, Anyang, China. *E-value* = 0.0001 was set to predict the cotton *TPS* family. The screened protein sequences were further confirmed according to their conserved domains using the online conserved domains analytical tool (http://www.ncbi.nlm.nih.gov/Structure/cdd/wrpsb.cgi).

### Phylogenetic analysis

The data of *TPS* protein sequences of *Arabidopsis* were downloaded from the *Arabidopsis* genome database (https://www.arabidopsis.org/). Rice *TPS* protein sequences were downloaded from the rice genome database (http://rice.plantbiology.msu.edu/). *Glycine max* (L.) Merrill *TPS* protein sequences were downloaded from Phytozome 11.0 (https://phytozome.jgi.doe.gov/pz/portal.html). *Zea mays TPS* protein sequences were downloaded from NCBI (http://www.ncbi.nlm.nih.gov/) and Phytozome 11.0 (https://phytozome.jgi.doe.gov/pz/portal.html/). *TPS* in *Arabidopsis*, rice, soybeans and *Zea mays* was named *AtTPS*, *OsTPS*, *GmTPS*, and *ZmTPS*, respectively. Multiple alignment of *TPS* proteins from the diploid cotton *G. raimondii* and *G. arboreum* L. and a tetraploid cotton *G. hirsutum* L. was performed using MEGA 5.1 [35]. Neighbor-joining method was used to construct gene trees and structure diagram.

### Basic structure of *TPS* gene family

ProParam online tool in ExPASy (http://www.expasy.org/tools/protparam.html) was used to analyze the basic physical and chemical properties of the protein sequences. Subcellular localization predictor (http://cello.life.nctu.edu.tw/) was used to predict subcellular localizations. The domain of the *TPS*s was analyzed using the conserved domains searcher tool (http://www.ncbi.nlm.nih.gov/Structure/cdd/wrpsb.cgi), and the motif analysis was carried out using MEME program (http://meme-suite.org/).

### Expression patterns analysis of cotton *TPSs* under stresses

The test cottons *G. raimondii*, *G. arboreum L. Shixiya* 1, and *G. hirsutum* L. TM-1were provided from the Institute of Cotton Research, Chinese Academy of Agricultural Sciences (CAAS, Anyang, China). Cotton plants were cultivated using the sand culture method [36]. Three seedlings at trefoil stage were exposed to low temperature (4.0 °C, 24 h), salt (150 mM NaCl, 24 h) and drought (sand moisture content 5.0 %), respectively. Then the roots, stems, and leaves were sampled and frozen in liquid nitrogen at −80 °C. Total RNA was extracted and reverse transcribed into cDNA. Primer Premier 5.0 (PREMIER Biosoft) was used to design fluorescent quantitative primers (Additional file 3: Table S1) for qRT-PCR. Fluorescent quantitative real-time polymerase chain reaction (qRT-PCR) was performed using *Histone-3* (Accession No. AF02471) as a reference gene. PCR reaction conditions and programs were set as follows: 94 °C for 30 s, 94 °C for 5.0 s, 55 °C for 34 s, and 72 °C for 34 s for a total of 40 cycles. Relative quantitative analysis of target genes was calculated with the 2^- Δ ΔCT^ method.

### Availability of supporting data

The data sets supporting the results of this article are included within the article and its additional files.

## Additional files

Additional file 1: Multiple alignment analysis of cotton *TPS*s (FAS 83 kb) Additional file 2: Figure S1. Motif sequences of cotton *TPS*s (DOCX 999 kb) Additional file 3: Table S1. Primers used for qRT-PCR (XLS 15 kb)

## Abbreviations

*GaTPSs*: genes in *G. arboreum* L. (AA)
*GhTPSs*: genes in *Gossypium hirsutum* L. (AADD)
*GrTPSs*: genes in *G. raimondii* (DD)
TPS: trehalose-6-phosphate synthase
