# Erratum to: Genome-wide Identification and analysis of the stress-resistance function of the TPS (Trehalose-6-Phosphate Synthase) gene family in cotton

**DOI:** 10.1186/s12863-016-0406-1

**Published:** 2016-07-05

**Authors:** Min Mu, Xu-Ke Lu, Jun-Juan Wang, De-Long Wang, Zu-Jun Yin, Shuai Wang, Wei-Li Fan, Wu-Wei Ye

**Affiliations:** State Key Laboratory of Cotton Biology/Institute of Cotton Research of Chinese Academy of Agricultural Sciences, Anyang, Henan 455000 China

Following publication of the original article in BMC Genetics [1], it was brought to our attention that the authors Min Mu and Xu-Ke contributed equally to this work.

Furthermore, the keywords “Rehalose-6-phosphase synthase (TPS) and Gossypium arboretum” are incorrect and should read: Trehalose-6-phosphase (TPS) and *Gossypium arboretum* L.

Also, in the “Methods-Expression patterns analysis of cotton TPSs under stresses” section, two extra blanks were introduced in “2- Δ ΔCT” in the last sentence. The sentence should read: Relative quantitative analysis of target genes was calculated with the 2-ΔΔCT method.

The original article has been updated with the changes listed above.
