# Supplementary figures and images for: Genome-wide Identification and analysis of the stress-resistance function of the TPS (Trehalose-6-Phosphate Synthase) gene family in cotton
Source: BMC Genet. 2016 Mar 18;17:54. doi: 10.1186/s12863-016-0360-y (PMC4797179; doi:10.1186/s12863-016-0360-y)

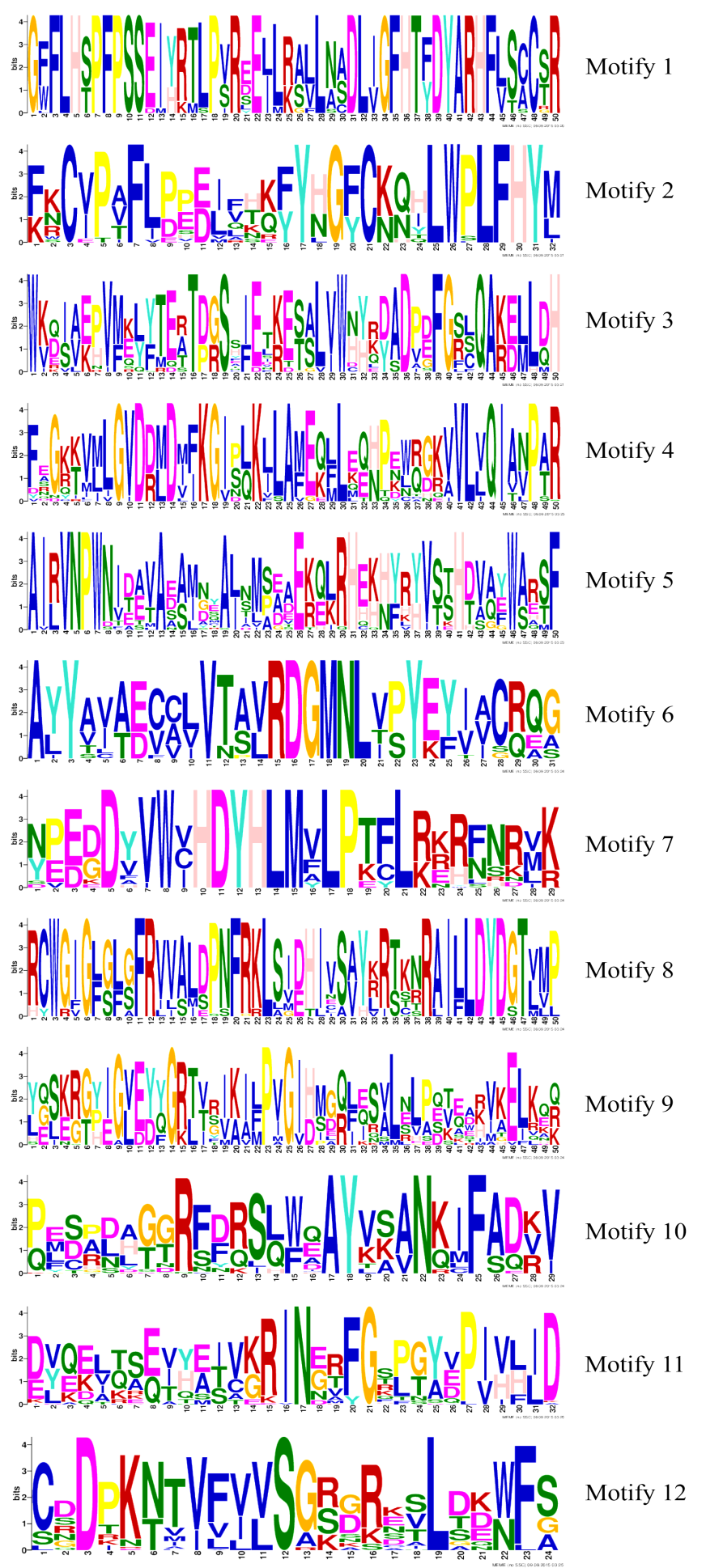


Additional file 2:Figure S1 Motif sequences of cotton *TPS*s

Supplement: Additional file 2: Figure S1. — Motif sequences of cotton TPSs (DOCX 999 kb) [file 12863_2016_360_MOESM2_ESM.docx]
